# Antiproliferative and Cytotoxic Activity of Xanthohumol and Its Non-Estrogenic Derivatives in Colon and Hepatocellular Carcinoma Cell Lines

**DOI:** 10.3390/ijms20051203

**Published:** 2019-03-09

**Authors:** Isabelle E. Logan, Cristobal L. Miranda, Malcolm B. Lowry, Claudia S. Maier, Jan F. Stevens, Adrian F. Gombart

**Affiliations:** 1 Department of Biochemistry and Biophysics, Linus Pauling Institute, Oregon State University, Corvallis, OR 97331, USA; logani@oregonstate.edu; 2 Department of Pharmaceutical Sciences, Linus Pauling Institute, Oregon State University, Corvallis, OR 97331, USA; Cristobal.Miranda@oregonstate.edu (C.L.M.); fred.stevens@oregonstate.edu (J.F.S.); 3 Department of Microbiology, Linus Pauling Institute, Oregon State University, Corvallis, OR 97331, USA; Malcolm.Lowry@oregonstate.edu; 4 Department of Chemistry, Oregon State University, Corvallis, OR 97331, USA; claudia.maier@oregonstate.edu; 5 Linus Pauling Institute, Department of Biochemistry and Biophysics, Oregon State University, Corvallis, OR 97331, USA

**Keywords:** xanthohumol, polyphenol, flavonoid, apoptosis, colon cancer, dihydroxanthohumol, tetrahydroxanthohumol, cell cycle, phytochemical, chemoprevention, dietary agent

## Abstract

Xanthohumol (XN), a prenylated flavonoid found in hops, inhibits growth in a variety of cancer cell lines; however, its use raises concerns as gut microbiota and the host’s hepatic cytochrome P450 enzymes metabolize it into the most potent phytoestrogen known, 8-prenylnaringenin (8-PN). The XN derivatives dihydroxanthohumol (DXN) and tetrahydroxanthohumol (TXN) are not metabolized into 8-PN and they show higher tissue concentrations in vivo compared with XN when orally administered to mice at the same dose. Here we show that DXN and TXN possess improved anti-proliferative activity compared with XN in two colon (HCT116, HT29) and two hepatocellular (HepG2, Huh7) carcinoma cell lines, as indicated by their respective IC_50_ values. Furthermore, XN, DXN, and TXN induce extensive apoptosis in all these carcinoma cell lines. Finally, TXN induces G_0_/G_1_ cell cycle arrest in the colon carcinoma cell line HT29. Our findings suggest that DXN and TXN could show promise as therapeutic agents against colorectal and liver cancer in preclinical studies without the drawback of metabolism into a phytoestrogen.

## 1. Introduction

First discovered at the turn of the twentieth century by Power et al., XN is a simple prenylated flavonoid found in the female inflorescences, or hop cones, of the hop plant *Humulus lupulus* [1]. Hops are used to add flavor, color, and bitterness to beer. Since hops are rarely used in a culinary setting, beer represents the main dietary source of XN. Extensive studies show XN inhibits cancer cell growth in vitro, and reduces weight gain and improves cognitive function in vivo [2,3,4,5,6,7,8,9]. Nevertheless, despite these positive effects, the use of XN raises concerns, as it was shown both in vitro and in vivo that XN is metabolized into 8-prenylnaringenin (8-PN), the most potent phytoestrogen currently known [10].

Synthesis of XN from phloracetophenone is inefficient [11]; therefore, XN is currently isolated from CO_2_-extracted hops, a byproduct of the hops industry, which provides a less expensive alternative to synthesis [2]. Because of the inexpensive production of XN and its purported health benefits, it is marketed as a dietary supplement. Preclinical animal studies show that XN is safe in high doses. Female BALB/c mice fed XN at 1000 mg/kg body weight for three weeks exhibited no adverse effects on major organ function and homoeostasis [12]. Furthermore, Sprague Dawley rats treated with 1000 mg/kg body weight per day by oral gavage showed only weak hepatotoxicity and poor development of mammary glands, neither of which are fatal [13]. In humans, an escalating dose study was performed in menopausal women using an extract from hops rich in XN. The results showed that the extract does not affect the sex hormones estradiol, follicle stimulating hormone, or luteinizing hormone, does not affect blood clotting, and caused no acute toxicity [14].

**Figure 1:**
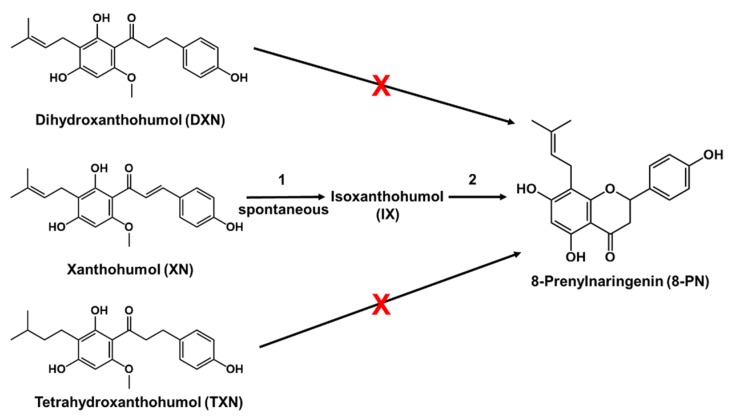
Structural comparison of dihydroxanthohumol (DXN), tetrahydroxanthohumol (TXN), xanthohumol (XN), and 8-prenylnaringenin (8-PN). Xanthohumol is converted to 8-prenylnaringenin in a two-step process. In step 1, xanthohumol spontaneously isomerizes to isoxanthohumol (IX). In step 2, isoxanthohumol is converted to 8-prenylnaringenin by the gut microbiome, or cytochrome P450 enzymes of the host [10,18]. XN can be converted into DXN by gut microbiota [19]. 8-PN formation is not possible with DXN or TXN. Figure adapted from [3].

Previous studies show that XN readily isomerizes to isoxanthohumol (IX), which is the first step in the metabolism of XN (Figure 1, pathway 1). This isomerization is enhanced by the high temperatures used during wort boiling [15]. It also occurs in the stomach, due to the acidic conditions encountered there [16]. After isomerization, the gut microbiome and the host’s hepatic cytochrome P450 enzymes metabolize IX into 8-PN [10]. A previous study showed *Eubacterium limosum* metabolizes IX via *O*-demethylation (Figure 1, pathway 2) [17]. Up to 34% of XN entering the gut can be converted to 8-PN; therefore, the microbiome plays a significant role in the formation of 8-PN from XN and IX. [18]. Our own research group recently discovered that *E. ramulus* metabolizes XN into DXN, and 8-PN into *O*-desmethylxanthohumol and *O*-desmethyl-α,β-dihydroxanthohumol [19]. On the other hand, both DXN and TXN differ from XN in that they lack the α,β-unsaturated ketone found in XN; therefore, interconversion to XN or IX is not metabolically possible [3]. This property prevents the formation of 8-PN, one of the most potent phytoestrogens known (Figure 1) [3,20,21]. Interestingly, 8-PN possesses anti-proliferative effects in vitro against the colon carcinoma cell line Caco-2, breast carcinoma line MCF-7, the melanoma line SK-MEL-28, and a Burkitt lymphoma cell line [21,22,23,24,25]. The estrogenic effects of 8-PN also show promising clinical results in treating postmenopausal symptoms, but these studies are usually short term and were not designed to assess the effect of potential cancer promoting properties [26]. In the host, 8-PN acts as a strong agonist for estrogen receptors located in cells throughout the body, which could increase one’s risk of developing hormone-dependent cancers [27,28]. Thus, its estrogenic activity is of concern and additional studies to assess the safety of 8-PN in long-term supplementation are needed [26].

In vitro, XN inhibits proliferation of breast, colon, hepatocellular, ovarian, pancreatic, prostate, and medullary thyroid cancer cell lines [5,9,29,30,31,32,33,34,35,36,37,38]. Treatment of four hepatocellular carcinoma cell lines—Huh-7, HepG2, Hep3B, and SK-Hep-1—with XN decreased both viability and colony forming ability [39]. XN also caused dose-dependent cytotoxic effects in colon carcinoma cell lines HT29, HCT15, and 40-16 [5,36,40]. Several apoptotic mechanisms were identified in the various cell types. In 40-16 cells, XN activates the death receptor and mitochondrial apoptosis pathway [36]; however, in HCT116 cells, XN induces apoptosis through PKA inhibition via upregulation of adenylate cyclase, while HT29 cells appeared insensitive to XN toxicity [41]. In the metastatic colon carcinoma line SW620, XN was proposed to act as a mitocan, because it induces apoptosis by impairing mitochondrial function [42]. On the other hand, in the hepatocellular carcinoma line HepG2, XN induces apoptosis through the NF-κB/p53-apoptosis signaling pathway [43].

Here we demonstrate the anti-proliferative and cytotoxic effects of XN in two human colon adenocarcinoma cell lines (HT29 and HCT116) and two human hepatocellular carcinoma cell lines (HepG2 and Huh7). Furthermore, we show for the first time that DXN and TXN possess similar effects, and in most cases, a lower half-maximal growth inhibitory concentration (IC_50_) than XN in these cell types. While XN and DXN do not appear to induce cell cycle arrest, HT29 cells treated with TXN arrest in the G_0_/G_1_ phase of the cell cycle. These findings demonstrate that the two non-estrogenic XN derivatives are attractive alternatives to test along with XN in future preclinical studies using mouse models of colorectal and hepatocellular carcinoma.

## 2. Results

### 2.1. XN, DXN, and TXN Inhibit Proliferation of Human Colon Adenocarcinoma and Liver Carcinoma Cell Lines

The half maximal inhibitory concentration (IC_50_) of DXN and TXN for two colon carcinoma and two hepatocellular carcinoma cell lines was determined using the SRB colorimetric assay for cytotoxicity screening, as previously described [44]. Table 1 lists the IC_50_ values experimentally determined for XN, DXN, and TXN in the colon carcinoma cell lines HCT116 and HT29, and the hepatocellular carcinoma cell lines HepG2 and Huh7. DXN and TXN (except HepG2) exhibited lower IC_50_ values than XN. For the cells most sensitive to DXN and TXN, the decrease in IC_50_ values was approximately 30% compared to XN (Table 1). In HepG2 cells, TXN was slightly less potent at inhibiting cell proliferation compared to XN, as indicated by a higher IC_50_ value (Table 1). Compared to DXN, TXN was more potent at inhibiting proliferation in Huh7 cells, as indicated by a lower IC_50_ value, whereas DXN was more potent in the other three cell lines (Table 1). HT29 was less sensitive (highest IC_50_ value) to XN treatment than HCT116, but showed a similar sensitivity to the derivatives DXN and TXN as HCT116 (Table 1).

### 2.2. XN and Derivatives Induce Apoptosis

**Figure 2:**
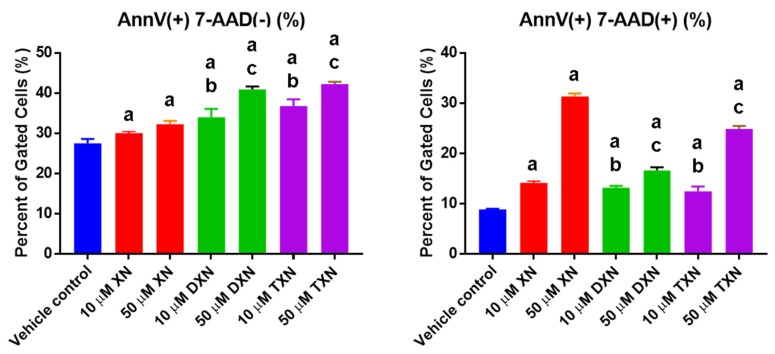
XN, DXN, and TXN induce apoptosis in HT29 cells. Cells were treated with each prenylated flavonoid at the concentration indicated (10 or 50 µM), and apoptosis was detected using an Annexin V assay. Externalization of phosphatidyl serine, an early marker of apoptosis, was detected by flow cytometry after staining with Annexin V-PE and 7-AAD. Annexin V(^+^) 7-AAD(^−^) cells are undergoing early apoptosis (**left panel**), and Annexin V(^+^) 7-AAD(^+^) are undergoing late apoptosis (**right panel**). Values are mean ± SD, *n* = 9 for each treatment. The letter “a” indicates a statistically significant difference from vehicle control, the letter “b” indicates a statistically significant difference from 10 µM XN, and the letter “c” indicates a statistically significant difference from 50 µM XN (*p* < 0.05). Statistical analysis was performed using one-way ANOVA with a Sidak’s post hoc test.

The Annexin V assay distinguishes the early apoptotic stage from the late necrotic state. Externalization of phosphatidyl serine, an early marker of apoptosis, was detected by flow cytometry after staining with Annexin V-PE and 7AAD. HT29 cells were treated for 18 h with either vehicle control, XN, DXN, or TXN. All compounds significantly induced apoptosis dose-dependently in these cells, compared to vehicle control (Figure 2). At 18 h post treatment, more DXN- and TXN-treated cells were in early apoptosis, whereas more XN-treated cells were in late apoptosis.

**Figure 3:**
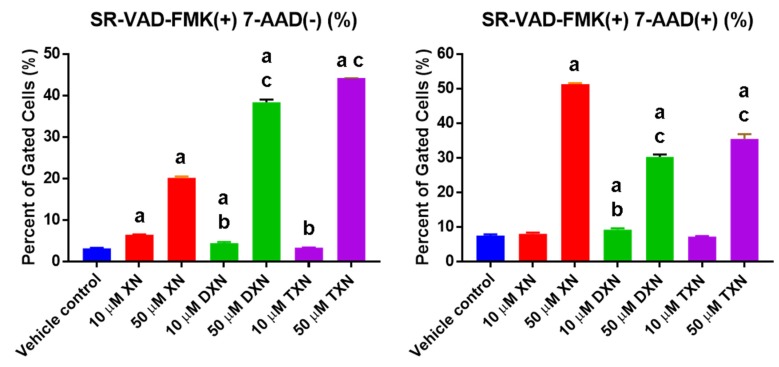
XN, DXN, and TXN induce caspase activation, cellular plasma membrane permeabilization, and cell death in HT29 cells. Cells were treated with each prenylated flavonoid at the concentration indicated (10 or 50 µM) for 18 h and apoptosis was detected using the multicaspase assay. Apoptosis was detected by flow cytometry after staining with SR-VAD-FMK and 7-AAD. SR-VAD-FMK (^+^) 7-AAD(^−^) cells are undergoing early- to mid-apoptosis (**left panel**), and SR-VAD-FMK (^+^) 7-AAD(^+^) are undergoing late apoptosis (**right panel**). Values are mean ± SD, *n* = 9 for each treatment. The letter “a” indicates a statistically significant difference from vehicle control, the letter “b” indicates a statistically significant difference from 10 µM XN, and the letter “c” indicates a statistically significant difference from 50 µM XN (*p* < 0.05). Statistical analysis was performed using one-way ANOVA with a Sidak’s post hoc test.

Induction of apoptosis was confirmed by measuring activation of caspase enzymes. In HT29 cells (Figure 3), treatment with XN and its derivatives for 18 h resulted in a dose-dependent increase in cells undergoing the early stages of apoptosis (SR-VAD-FMK(^+^) 7-AAD(^−^)), indicating caspase activation without membrane alterations (early- to mid-stage apoptosis). In addition, there was a statistically significant increase in doubly stained SR-VAD-FMK(^+^) 7-AAD(^+^) cells, indicating cells in late apoptosis (Figure 3).

At 18 h post treatment, more DXN- and TXN-treated cells were in early- to mid-apoptosis, whereas more XN-treated cells were in late apoptosis. Taken together, the data from both assays suggest that XN may promote a slightly more rapid apoptosis than DXN or TXN in HT29 cells.

**Figure 4:**
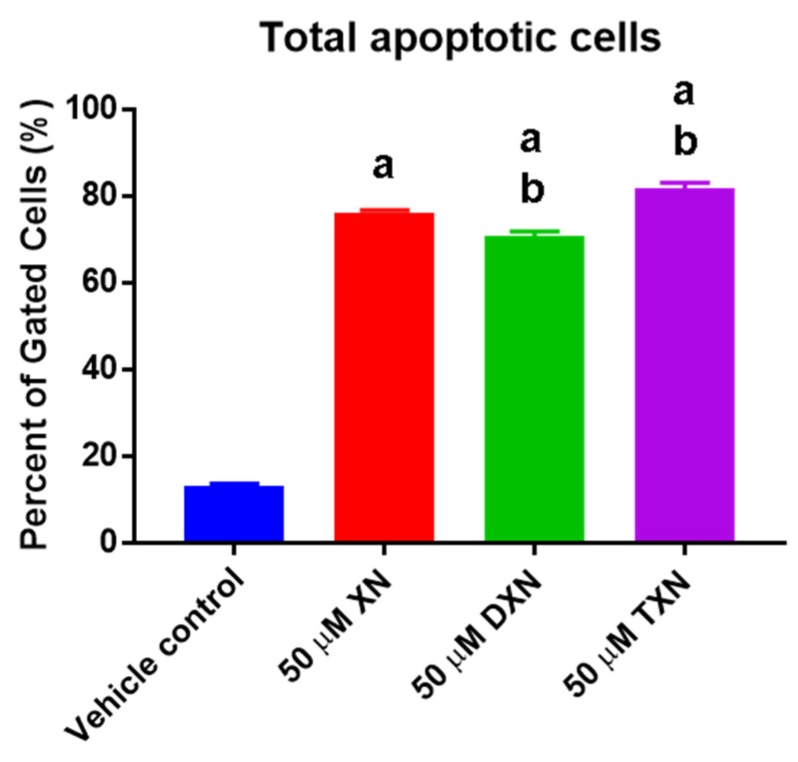
Total percentage of apoptotic and dead cells. Values are mean ± SD, *n* = 9 for each condition. The letter “a” indicates a statistically significant difference from vehicle control, the letter “b” indicates a statistically significant difference from 50 µM XN (*p* < 0.05). Statistical analysis was performed using one-way ANOVA with a Sidak’s post hoc test.

Quantifying the total number of dead and apoptotic cells from the caspase assay showed that DXN induced slightly less apoptosis (−7.2%), while TXN induced slightly greater apoptosis (+7.1%) than XN (Figure 4).

### 2.3. TXN Induces a G_1_ Cell Cycle Arrest in HT29 Cells

**Figure 5:**
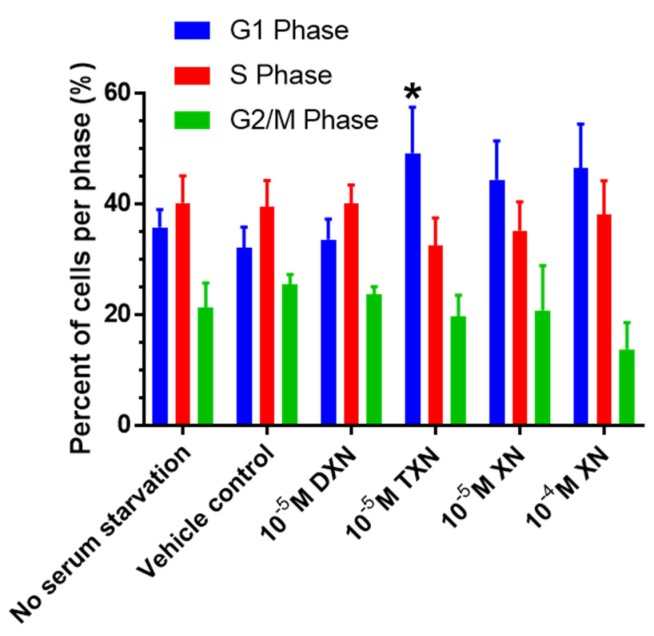
Cell cycle analysis using HT29 cells and propidium iodide, values are mean ± SD, *n* = 9 for each treatment. An asterisk indicates a statistically significant difference from vehicle control (*p* < 0.05). Statistical analysis was performed using one-way ANOVA with a Sidak’s post hoc test.

We treated HT29 cells for 24 h following cell cycle synchronization by serum starvation, and performed cycle analysis with propidium iodide staining. Results showed that only TXN induced a statistically significant cell cycle arrest in these cells, as indicated by an increased accumulation of cells in the G_1_ phase (Figure 5). All prenylated flavonoids induced cell apoptosis, as indicated by a sub G_0_/G_1_ population of 82%, 60%, and 45% in the cells treated with XN, DXN, and TXN, respectively.

## 3. Discussion

In the United States, colorectal cancer is the third most common cause of cancer-related death [45] and it is the second leading cause of cancer-related deaths in the developed world [46,47]. The incidence of liver cancer has tripled since 1980, mortality has doubled during that time, and it is the fifth most common cause of cancer death in the United States. [45]. For both cancers, it is imperative to discover new compounds for prevention and treatment. The prenylated flavonoid XN was previously shown to inhibit the growth of the colon cancer cell lines HT-29 and 40-16 in a dose-dependent manner [5,36]. XN also induced apoptosis in the liver carcinoma cell lines HepG2 and Huh7 in a dose-dependent manner [39]. In the current study, we determined IC_50_ values of 40.8 ± 1.4, 50.2 ± 1.4, 25.4 ± 1.1, and 37.2 ± 1.5 µM for XN, in HCT116, HT29, HepG2, and Huh7 cell lines, respectively. These results indicate that both hepatocellular and colon carcinoma cells are sensitive to growth inhibition by XN. The IC_50_ values determined for HepG2 and Huh7 cells in this study were, on average, three-fold lower than those calculated for the hepatocellular carcinoma cells HA22T/VGH and Hep3B, but still in the micromolar range [48]. In this study, the IC_50_ values determined for HT29 and HCT116 were higher than those determined for the HCT15 and 40-16 colon carcinoma cell lines [36,40]. The differences in in vitro sensitivity with the different cell lines could have implications for preventative treatments, but this would require experiments with in vivo xenograft models in mice to determine. It is interesting to note that Shikata et al. (2017) showed the sensitivity of the HCT116 and SW480 colon cancer cell lines to XN, with IC_50_ values in the micromolar range in vitro, reflected by their in vivo sensitivity in a xenograft model [41]. While we found that HT29 and HCT116 cells were both sensitive to treatment with XN and its derivatives, HT29 was less sensitive than HCT116. This is consistent with a previous study by Shikata et al. (2017) that determined HT29 cells were insensitive compared to HCT116 cells by measuring the sub-G_1_ population after treatment [41]. Although considered insensitive, HT29 was still affected by XN treatment, but required a higher dose before showing cytotoxicity. In their cell cycle assay, the highest dose was 30 μM, whereas we used 50 μM. It is possible that Shikata et al. (2017) would have observed an increased sub-G_1_ population if they had used this dose. In addition, our study concurs with those performed previously by others, who observed that HT29 cells are sensitive to XN treatment, but were the least sensitive of several cancer cell lines tested as reflected by a higher IC_50_ [5,49,50]. It is possible to attribute the differences between studies to experimental design, cell density, or different culture conditions. Taken together, the findings indicate that XN is cytotoxic to various colon and liver cancer cell lines. 

XN is metabolized into the potent phytoestrogen 8-PN via conversion into IX (Figure 1). We recently demonstrated that DXN and TXN are non-estrogenic, as these derivatives lack the α,β-unsaturated ketone found in XN; therefore, interconversion to XN or IX is not metabolically possible [3]. This property prevents the formation of 8-PN [3,20,21]. Like XN, DXN possesses anti-proliferative activity in HT29 colon cancer and MCF7 breast cancer cell lines [51,52]. The IC_50_ value of DXN in HT29 cells was previously determined to range from 12 to 74 µM [51,52]. In the current study, we calculated a comparable IC_50_ value of 31.4 ± 1.1 µM. We observed that in all the cell lines tested, both DXN and TXN inhibited cellular growth, as well as XN. For most cell types, DXN and TXN were slightly more potent than XN. Out of the three compounds tested, TXN was the only compound that induced cell cycle arrest in the G_1_ phase. 

While all prenylated flavonoids tested induced apoptosis in HT29 cells, XN appeared to increase the rate of apoptosis. After 18 h, cells treated with DXN or TXN showed a slightly greater percentage undergoing early- to mid-apoptosis, and a slightly lower percentage of cells undergoing late apoptosis, compared to XN treatment. Nevertheless, when HT29 cells were treated with a 50 µM concentration of the prenylated flavonoids, the total number of dead or apoptotic cells was slightly greater in HT29 cells treated with TXN. Taken together, our data suggest that XN and its derivatives inhibit cancer cell proliferation through a molecular mechanism that includes caspase-mediated apoptosis.

Oral consumption of XN and its derivatives is expected to expose the intestinal cell layer to the highest concentrations. After initial uptake in the intestine, XN is transported to the liver through the portal vein. In the liver, XN is glucuronidated [53,54]. This conjugation significantly lowers the amount of XN found in other tissues [53,55,56]. In a study of metabolic syndrome and obesity, our research group observed that when added to the diet (30 mg/kg body weight), the concentrations of XN, DXN, and TXN were highest in the liver, with DXN and TXN 5- to 7-fold higher than total XN, which included conjugated forms and IX [3]. The tissue levels of XN and its derivatives ranged from 0.3–3.0 μM. We would expect much higher concentrations in the colon. In this same study, we noted that levels of DXN and TXN did not induce markers of hepatotoxicity, i.e., plasma aspartate aminotransferase (AST) or alanine aminotransferase (ALT), in control or treated mice [3]. In colon cancer xenograft models with mice, Shikata et al. (2017) injected XN IP daily for 17 days and observed a dramatic and significant reduction in tumor growth in SW480 and about a 2–3-fold reduction in tumor volume for HCT116 at doses of 1, 15, and 30 mg/kg body weight. They observed no changes in mouse body weight. Furthermore, administration of XN in xenograft models of breast, leukemia, prostate, pancreatic, gastric, and cholangiocarcinoma cells inhibits tumor growth [29,41,57,58,59,60,61,62,63]. Taken together, these data suggest that XN and its derivatives show promise in future studies. Because of its well-established anti-proliferative effects in cell culture and safety in preclinical studies, XN and its non-estrogenic derivatives are reasonable candidates for preclinical studies of colorectal and liver cancer, which is relatively untested even for XN, since the discovery of its anti-proliferative effects 20 years ago [5].

In future work, we will test XN, DXN, and TXN as potential treatments of colorectal and hepatocellular carcinoma using mouse models and further elucidate the molecular mechanisms by which these derivatives mediate their effects. Our findings suggest that these plant-derived molecules show promise as potential anticancer compounds.

## 4. Materials and Methods

### 4.1. Cell Culture

The human colon adenocarcinoma cell lines HT 29 and HCT116 were provided by Dr. H. Phillip Koeffler (Cedars-Sinai Medical Center, Los Angeles, CA, USA). The human liver carcinoma cell line HepG2 was provided by Dr. Tori Hagen (Oregon State University, Corvallis, OR, USA). We purchased Huh7 from the American Type Tissue Collection (ATCC, Manassas, VA, USA). The other cancer cell lines were originally purchased from ATCC. All cancer cell lines were grown and maintained in Dulbecco’s Modified Eagle’s Medium (DMEM) without sodium pyruvate (Corning, Manassas, VA, USA) supplemented with 10% fetal bovine serum (Hyclone, Logan, UT, USA) and 1% penicillin–streptomycin (Gibco, Grand Island, NY, USA). All cell types were maintained in 5% CO_2_ at 37 °C with humidity.

### 4.2. Treatments

XN and TXN were provided by Hopsteiner Inc., while DXN was synthesized as described previously [3]. The purity and identity of the prenylated flavonoids XN, DXN, and TXN, were determined by mass and NMR spectrometry [3]. All prenylated flavonoids were dissolved in 100% ethanol and added to the culture medium at a final concentration of 1–100 µmol/L, depending on desired treatment. Confluency of cells was approximately 50–70% at time of treatment. As a vehicle control, ethanol was added to culture medium equivalent to prenylated flavonoid treatment. Final concentration of ethanol did not exceed 0.1%.

### 4.3. Cellular Proliferation

Cells were seeded into 96-well plates (15,000 cells per well). After 24 h, cells were treated with either XN, DXN, or TXN, as described above. Cell viability was evaluated using a sulforhodamine B (SRB) assay (Sigma-Aldrich, St. Louis, MO, USA), as described previously [44]. Absorbance of dye was measured at a wavelength of 565 nm, using a Molecular Devices SpectraMax (Molecular Devices, Sunnyvale, CA, USA) plate reader.

### 4.4. Flow Cytometry

#### 4.4.1. Apoptosis

At various times after treatment with XN, DXN, or TXN, adherent and floating cells were collected for analysis. Apoptosis was assessed using flow cytometry-based annexin V (Invitrogen™. eBioscience™ Annexin V Apoptosis Detection Kit PE, Thermo Fisher, Waltham, MA, USA) and a multicaspase assay kit (MilliporeSigma, Burlington, MA, USA). The annexin V assay is based on measurement of externalization of phosphatidyl serine, a common characteristic of cells undergoing apoptosis. The multicaspase assay is based on measurement of caspase enzymes activated during apoptosis. Cells were trypsinized, washed in Dulbecco’s Phosphate Buffered Saline (D-PBS), and stained with Annexin V, sulforhodamine-valyl-alanyl-aspartyl-fluoromethyl-ketone (SR-VAD-FMK), and 7-amino-actinomycin D (7AAD) according to the manufacturer’s instructions. Cell populations were quantified using a Guava personal cytometer (Guava Technologies, Burlingame, CA, USA).

#### 4.4.2. Cell Cycle Analysis

At various time points after treatment with XN, DXN, or TXN, adherent and floating cells were collected and fixed in 70% ethanol at one million cells per aliquot. Samples were centrifuged at 500× *g*, washed with PBS, and re-suspended in cellular DNA staining solution containing 40 mg/mL propidium iodide (Sigma-Aldrich, St. Louis, MO, USA) and 100 mg/mL RNase A (Sigma-Aldrich) in PBS. After a 15-min incubation at room temperature, cell populations were quantified using a Guava personal cytometer (Guava Technologies). 

### 4.5. Statistical Analysis

Statistical analyses were performed with the GraphPad Prism 7 software (GraphPad Software, Sand Diego, CA, USA). Group differences were assessed with a one-way ANOVA, followed by a Sidak’s post hoc test. The Sidak’s post hoc test was used to compare every mean with either the control mean, or XN mean. Statistical significance was set at *p* ≤ 0.05.

## Figures and Tables

**Table 1:** XN, DXN, and TXN inhibit proliferation of human colon adenocarcinoma (HCT116 and HT29) and liver carcinoma (HepG2 and Huh7) cell lines. Mean IC_50_ values (±SD) were calculated from cell growth curves (*n* = 5, Figure S1).

Cell Type	IC_50_ (µM)					
	XN	SD	DXN	SD	TXN	SD
**HCT116**	40.8	1.4	28.7 ^a^	1.0	34.0 ^b^	1.3
**HT29**	50.2	1.4	31.4 ^a^	1.1	34.9 ^b^	1.1
**HepG2**	25.4	1.1	21.7 ^a^	1.1	27.1 ^b^	1.1
**Huh7**	37.2	1.5	32.5 ^a^	1.3	26.5 ^b^	1.1

^a^ Statistically significantly different from XN treatment *p* < 0.05); ^b^ Statistically significantly different from XN and DXN (*p* < 0.05). Statistical analysis was performed using one-way ANOVA with a Sidak’s post hoc test.

## Supplementary Materials

Supplementary materials can be found at https://www.mdpi.com/1422-0067/20/5/1203/s1.

## Abbreviations

XN	Xanthohumol
DXN	Dihydroxanthohumol
TXN	Tetrahydroxanthohumol
8-PN	8-Prenylnaringenin
IX	Isoxanthohumol
7AAD	7-amino-actinomycin D
SR-VAD-FMK	Sulforhodamine-valyl-alanyl-aspartyl-fluoromethyl-ketone
DMEM	Dulbecco’s Modified Eagle Media
D-PBS	Dulbecco’s Phosphate-Buffered Saline
