# Supplementary material for: Antiproliferative and Cytotoxic Activity of Xanthohumol and Its Non-Estrogenic Derivatives in Colon and Hepatocellular Carcinoma Cell Lines
Source: Int J Mol Sci. 2019 Mar 9;20(5):1203. doi: 10.3390/ijms20051203 (PMC6429097; doi:10.3390/ijms20051203)
Supplement: Supplementary file 1 [file ijms-20-01203-s001.pdf]

International Journal of
Molecular Sciences

MDPI

Supplementary Materials

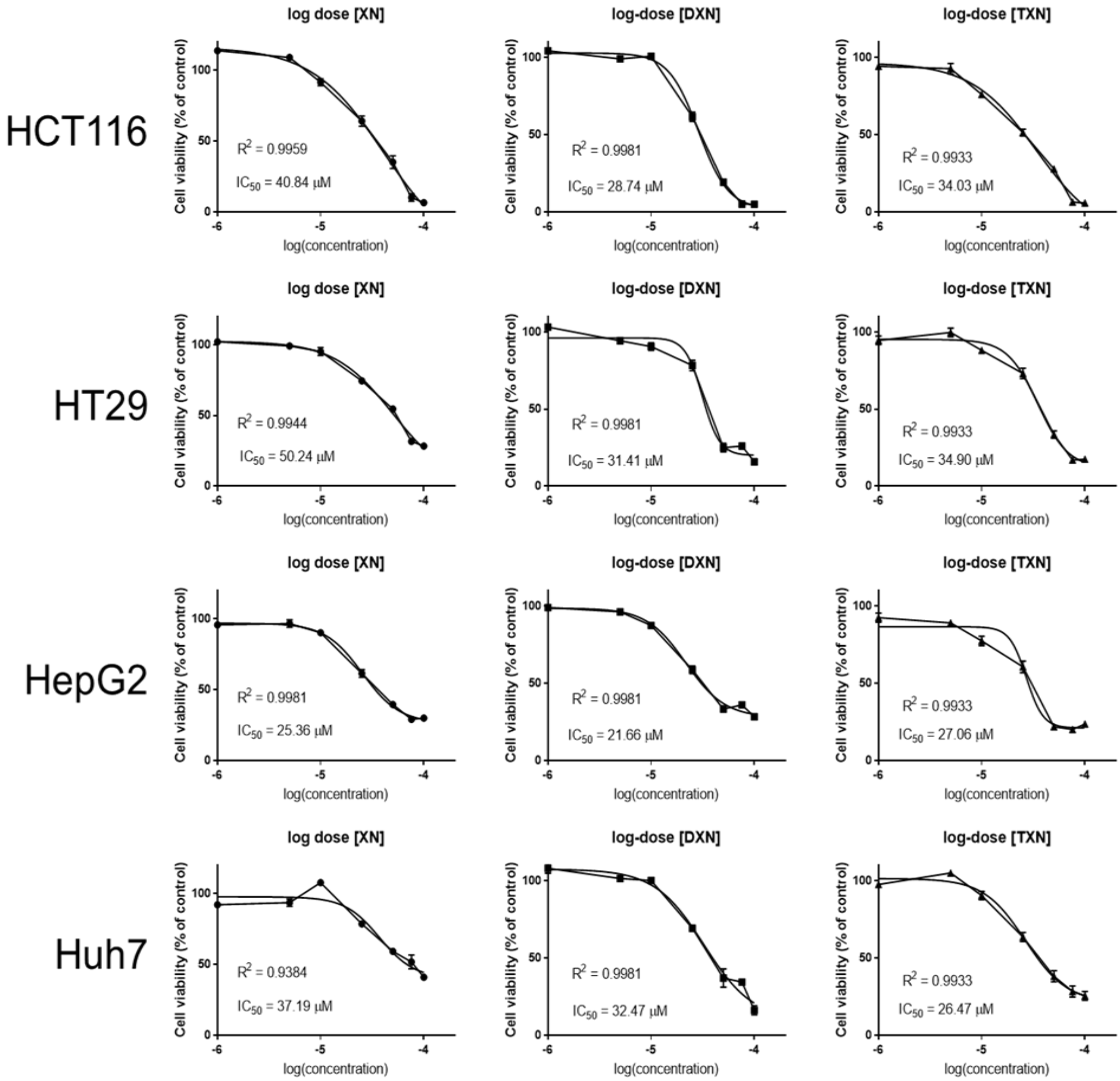

Figure S1. Comprehensive SRB assay results indicating cell viability curves, $n = 5$ per dose.
